# Fuzzy Representation of Principal’s Preferences in Inspire Negotiation Support System

**DOI:** 10.3390/e23080981

**Published:** 2021-07-29

**Authors:** Krzysztof Piasecki, Ewa Roszkowska, Tomasz Wachowicz, Marzena Filipowicz-Chomko, Anna Łyczkowska-Hanćkowiak

**Affiliations:** 1 Institute of Economics and Finance, WSB University in Poznan, 60-005 Poznań, Poland; krzysztof.piasecki@wsb.poznan.pl (K.P.); anna.lyczkowska-hanckowiak@wsb.poznan.pl (A.Ł.-H.); 2 Faculty of Economics and Finance, University of Białystok, 15-062 Białystok, Poland; 3 Department of Operations Research, University of Economics in Katowice, 40-287 Katowice, Poland; tomasz.wachowicz@uekat.pl; 4 Faculty of Computer Science, Bialystok University of Technology, 15-351 Białystok, Poland; m.filipowicz@pb.edu.pl

**Keywords:** Inspire, negotiation, preferences, fuzzy utility, fuzzy preferences

## Abstract

We consider the negotiation problem, in which an agent negotiates on behalf of a principal. Our considerations are focused on the Inspire negotiation support system in which the principal’s preferences are visualised by circles. In this way, the principal describes the importance of each negotiation issue and the relative utility of each considered option. The paper proposes how this preference information may be implemented by the agent for determining a scoring function used to support decisions throughout the negotiation process. The starting point of our considerations is a discussion regarding the visualisation of the principal’s preferences. We assume here that the importance of each issue and the utility of each option increases with the size of the circle representing them. The imprecise meaning of the notion of “circle size” implies that in a considered case, the utility of an option should be evaluated by a fuzzy number. The proposed utility fuzzification is justified by a simple analysis of results obtained from the empirical prenegotiation experiment. A novel method is proposed to determine trapezoidal fuzzy numbers, which evaluates an option’s utility using a series of answers given by the participants of the experiment. The utilities obtained this way are applied to determine the fuzzy scoring function for an agent. By determining such a common generalised fuzzy scoring system, our approach helps agents handle the differences in human cognitive processes associated with understanding the principal’s preferences. This work is the first approach to fuzzification of the preferences in the Inspire negotiation support system.

## 1. Introduction

Negotiation analysis is a subdiscipline of decision theory, which is focused on developing tools and techniques for efficient negotiation support [1]. An underlying element of negotiation analysis is the negotiation template and its rating system. The former describes the negotiation problem’s structure, while the latter—the negotiator’s preferences over its elements. Based on all parties’ scoring systems, individual or joint support may be offered to the negotiators either by third parties or software and electronic support systems [2,3,4], e.g., in selecting offers for bargaining, comparisons of concessions, and evaluating the negotiation agreement.

Inspire was the first web-based negotiation support system developed by Gregory Kersten in the early 1990s [2]. This system is used to support activities conducted in each negotiation phase: prenegotiation, actual negotiation, and post-settlement. Inspire can also be used as a negotiation simulator and training and teaching tool. It has been presented to students from more than 50 countries at many universities as part of regular courses such as information systems, decision-making, and negotiations [5]. Inspire is, for now, the software system most widely used in negotiation studies and research [6]. Many researchers use the experimental data from Inspire to study computer support in negotiation [5,7,8], behavioural aspects of decision making in negotiation [7,9,10], preference analysis [7,9,11,12], and cross-cultural differences in decision making [13] are among others.

In the Inspire system, the negotiation experiments are conducted using a protocol for representative negotiations [14,15]. In representative negotiations, the agents (negotiators) negotiate on behalf of their principals. The principals use agents as they may have better negotiation skills and knowledge to negotiate efficient contracts for them [16,17]. However, a new problem arises, i.e., the construction of scoring systems by agents that accurately represent the principals’ preferences. Such systems can be determined accurately provided the principal precisely imparts her preferences to an agent beforehand. Unfortunately, principals may not be able to impart precise information on their goals and priorities to their agents due to a lack of decision-making skills, cognitive limitations, or low numerical intelligence. In such a case, the verbal description of preferences may be accompanied by various visualisations [18]. Some examples of determining preferences with the use of popular visualisation techniques are presented in [19,20,21]. Miettinen [18] and MacDonald-Ross [22] discuss the following graphic formats of graphic presentation: bar charts, pie charts, cartograms, scatter plots, and or similar plane figures in different sizes.

Out of the visualisation methods mentioned above, circles are one of the most common [12]. They are popular in the negotiation support systems and were used first by Kersten in his Inspire system [13] and later in eNego [23]. Using circles to represent the preferences seems to be quite convenient and cognitively easy for principals. However, it may raise interpretational problems as the circles are two-dimensional [12,24,25]. This way of preference representation may also be linked to the imprecise perception of preferences by the principals themselves. Therefore, the principal’s preferences should be expressed in imprecise terms. A commonly accepted model of an imprecise term is the fuzzy set [26].

The fuzzy sets are widely used to deal with imprecise or vague judgments and incomplete information in the negotiation process. The negotiation support systems based on fuzzy logic [27,28,29], fuzzy negotiation scoring systems [30,31,32,33,34], fuzzy negotiation strategies [35,36], and fuzzy protocols [37,38] were proposed. In some of the papers, the preferences of the system’s user (who can be the principal or their agent) were expressed using the linguistic scale discussed in [31,39,40]. In line with Zadeh [41,42,43], the ratings corresponding to the linguistic terms were represented by fuzzy numbers (FN). However, this linguistic scale and its links to the numbers were given ex-cathedra, i.e., arbitrarily established by the scale designers. Consequently, the ability to express the principal’s preferences is limited, first—because the scale itself is small, and, second—because the meaning of the linguistic terms may have different cardinal consequences for the negotiator than the ones represented by predefined fuzzy numbers.

The problem of determining the scoring system by an agent for his principal that would implement a fuzzy approach and comprehensively address the nuances of the preferences expressed by the latter in a visual form has not been previously studied. As the circles drawn by the principal may be interpreted in different ways, the main goal of this paper is to propose the scoring function that considers the potential variety of possible interpretations of the circles drawn by the principal. We propose a different approach to determining the fuzzy scoring system than it can be classically made by implementing typical fuzzy multiple criteria decision aiding techniques by an individual agent, e.g., the fuzzy AHP method [44]. We assume that the agent uses the broader opinion of how the principal’s preferences may be interpreted, e.g., obtained experimentally from the surveys. As these interpretations may differ due to the respondents’ cognitive capabilities and information processing styles (see [12,45]), they are then aggregated into a form of a fuzzy scoring system. The trapezoidal fuzzy numbers are used to build the fuzzy scoring function to convey the ample information provided by different respondents most aptly. The key merit of such a fuzzy scoring system is that it allows the agent to operate with the fuzzy scale that is specific to the situational context of the negotiation problem under consideration and its interpretation by a wider group of interpreters. This avoids setting up the scoring system using a single individual interpretation only that may be biased due to specific cognitive limitations of a particular respondent or the agent. Simultaneously, respondents’ responses are not aggregated into a scalar form, which would result in losing the nuances of interpretation of the principal’s preferences.

Summing up, the paper makes an impact in the analysis of preferences in representative negotiations in the following aspects:

(i) we discuss the problem of interpretation by an agent of the principal preferences visualised imprecise by circles;
(ii) we design a new procedure for building a fuzzy scoring system by an agent using simultaneous recommendations provided by many independent interpreters;
(iii) we identify some problems with an evaluation of preferential information by such interpreters linked with the normalisation procedures.

The paper is organised in the following way. Selected facts about FNs are outlined in Section 2. Section 3 presents the notions of the negotiation template and scoring function. Moreover, in this section, we discuss Kersten’s problem of visualisation of the principal’s preferences [12]. Section 4 briefly describes the prenegotiation experiment linked to the Inspire negotiation case [2]. Using results obtained in this experiment, we justify a fuzzification of a utility value. Section 5 presents some propositions of fuzzy scoring function related to Kersten’s problem of preferences visualisation. In Section 6, we provide a discussion of the advantages of using our fuzzy approach to better interpret the principal’s preferences and its use in a selection of negotiation offers in the bargaining process. Section 7 concludes the article, summarises the main findings of this research, and proposes some future research directions.

## 2. Fuzzy Numbers—Selected Facts

An imprecise quantity is a family of real numbers belonging to it at a varying degree. An imprecise number is FN, defined as a convex fuzzy set in the real line ℝ. The most general definition of a fuzzy number was given by Dubois and Prade [26].

A particular case of fuzzy numbers is trapezoidal fuzzy numbers (TrFN). Due to their simplicity and ease of performing operations, they are often used in real-life applications. A suitable definition of trapezoidal fuzzy numbers is given in [46]:

**Definition** **1.** *For any non-decreasing sequence* (a,b,c,d)⊂ℝ *, a trapezoidal fuzzy number is a fuzzy set* T=Tr(a,b,c,d) *defined by its membership functions* $\mu_{T}\in {\left[0,1\right]}^{\mathbb{R}}$ *in the following way*

(1) $$\mu_{T}\left(x\right)=\mu_{Tr}\left(x|a,b,c,d\right)=\{\begin{array}{c} \;0,\;x\notin \left[a,d\right],\\ \frac{x-a}{b-a},\;x\in [a,b[,\\ 1,\;x\in \left[b,c\right],\\ \frac{x-d}{c-d},\;x\in ]c,d]. \end{array}$$

From the point of view of a multi-valued logic, the value $\mu_{T}\left(x\right)$ is interpreted as the truth value of the sentence “number T is equal to x∈ℝ”. The space of all TrFNs is denoted by the symbol $F_{Tr}$.

In line with Zadeh’s Extension Principle [41,42,43], the addition ⊕ of TrFNs is determined in such a way that for any pair $\left(Tr\left(a,b,c,d\right),Tr\left(e,f,g,h\right)\right)\in F_{Tr}^{2}$ we get their sum

(2) Tr(a,b,c,d)⊕Tr(e,f,g,h)=Tr(a+e,b+f,c+g,d+h)

Let us consider the pair $\left(K,\;ℒ\right)\in F_{Tr}^{2}$ represented by the pair $\left(\mu_{K},\mu_{L}\right)\in {\left({\left[0,1\right]}^{\mathbb{R}}\right)}^{2}$ of their membership functions. On the space $F_{Tr}$, we introduce the relation K.GE.ℒ, which reads:

(3) TrFN K is greater than or equal to TrFN ℒ.

Orlovsky [47] shows that in agreement with Zadeh’s Extension Principle, this relation is a fuzzy preorder [GE] described on $F_{Tr}^{2}$ by its membership function $\nu_{\left[GE\right]}\in {\left[0,1\right]}^{F_{Tr}^{2}}$ determined as follows

(4) $$\nu_{\left[GE\right]}\left(K,\;ℒ\right)=\sup\left\{\min\left\{\mu_{K}\left(x\right),\mu_{L}\left(y\right)\right\}:x\geq y\right\}.$$

In agreement with the above, for any TrFNs $Tr\left(a,b,c,d\right),\;Tr\left(e,f,g,h\right)\in F_{Tr}$ we have

(5) $$\nu_{\left[GE\right]}\left(Tr\left(a,b,c,d\right),\;Tr\left(e,f,g,h\right)\right)=\{\begin{array}{c} 0,\;0<e-d,\\ \frac{e-d}{\left(c-d\right)-\left(f-e\right)},\;e-d\leq \;0<f-c.\\ 1,\;c\geq f \end{array}$$

For any pair $\left(K,\;ℒ\right)\in F_{Tr}^{2}$, the value $\nu_{\left[GE\right]}\left(K,ℒ\right)$ is interpreted as the truth value of the sentence (3).

The use of the relation [GE] allows determining the impact of the imprecision of the compared TrFNs on their comparison. For example, the preorder [GE] is applied for decision making in risk management [48], candidate selection for the job [49], supplier selection [50], and investment recommendations [51].

Determining the ranking of TrFN is very important in fuzzy decision making. In the literature, several ranking methods are presented based on preference relation [52], similarity measures [53], area [54], integral value [55], distance measure [56], among others. However, no single technique is considered universal [57,58]. In the majority of situations, decision makers simplify the ranking method. Therefore, they use the concept of the defuzzification technique [59]. Unfortunately, the defuzzification makes the fuzzy preorder [GE] to be substituted by crisp relation “greater than or equal to”, determined on ${\mathbb{R}}^{2}.$ Consequently, we lose the ability to explain what is the impact of the imprecision of TrFNs on the result of their comparison. The defuzzification may give the false perception of operating with precise and sound information, which in fact changes the true picture of the problem under consideration [39,60], and may have negative consequences for decision making. For this reason, we will stay with the fuzzy preorder [GE] when comparing TrFNs.

For any set $A\subset F_{Tr}$, we can determine the fuzzy set maxA of its non-dominated elements. In line with Zadeh’s Extension Principle, the fuzzy set maxA is described by its membership function $\psi_{\max A}\in {\left[0,1\right]}^{A}$ given by the identity

(6) $$\psi_{\max A}\left(K\right)=\min\left\{\nu_{\left[GE\right]}\left(K,ℒ\right):\;ℒ\in A\right\}.$$

## 3. Negotiation Template and Scoring Function

Negotiation analysis aims at supporting the negotiators (principals themselves or their agents) in achieving fair and mutually satisfying agreements. To this end, it suggests the parties recognise the structure of the negotiation problem and declare their preferences formally within the prenegotiation preparation phase (see [1,61,62]).

The structure of the negotiation problem, called a negotiation template, defines the issues to be negotiated and the sets of their feasible resolution levels (options). Formally, it can be described using the ordered pair T=(ℱ,X), where $ℱ={\left(f_{i}\right)}_{i=1}^{n}$ is a sequence of negotiation issues $f_{i}$, and $X={\left(X_{i}\right)}_{i=1}^{n}$ is a sequence of options lists $X_{i}$ related to issue $f_{i}$. Each options list $X_{i}$ may be considered as the sequence $X_{i}={\left(x_{i,j}\right)}_{j=1}^{m_{i}}$ of options. With the template agreed by the parties and defined formally as T, the set ℙ of feasible negotiation offers ${\bar{P}}_{p}$ may be defined as

(7) $$\mathbb{P}=X_{1}\times X_{2}\times \ldots \times X_{n}∋\bar{P_{p}}=\left(x_{1,j\left(1,p\right)},\ldots ,x_{n,j\left(n,p\right)}\right)$$

where $x_{i,j\left(i,p\right)}\in X_{i}$, denotes an option of ith issue used to build the package $\bar{P_{p}}$.

In the first step of prenegotiation preparation, the principal is asked to express her preferences over the elements of the template T=(ℱ,X). In general, we assume that the preferences are additive. Then each negotiation package is evaluated by use of scoring function $S:\mathbb{P}\to {\mathbb{R}}_{0}^{+}$ determined by the identity

(8) $$S\left(\bar{P_{p}}\right)=S\left(x_{1,j\left(1,p\right)},x_{2,j\left(2,p\right)},\ldots ,x_{n,j\left(n,p\right)}\right)=\sum_{i=1}^{n}U\left(x_{i,j\left(i,p\right)}\right),$$

where the symbol $U\left(x_{i,j\left(i,p\right)}\right)$ denotes a utility of option $x_{i,j\left(i,p\right)}$.

The pair ⟨ℙ, S⟩ is called the scoring system.

In the literature on the subject, we find an extensive discussion on the use of utility to describe the principals’ preferences [1,63,64]. In the Inspire system, the users determine the scoring systems through the protocol that implements a hybrid conjoint measurement algorithm [65]. We will illustrate all our considerations with the help of an example of the negotiation problem originally presented in Inspire [2]. This is a well-known case study that has been discussed in many papers [8,10,12,24].

**Example** **1.** *We consider a negotiation in which the agents of a musician (Fado) and a broadcasting company (Mosico) talk over the terms of a potential contract [2]* *. The negotiation template is defined using four issues, each having a predefined list of options that allow to build 240 various offers (see Table 1* *).*

In the Inspire system, the principal visualises her preferences on the template T by means of circles C(ϕ) of various radii ϕ unknown to the agent [2]. The principal can draw any circle belonging to the family

(9) $$O=\left\{C\left(\phi \right):\;\phi \in {\mathbb{R}}_{0}^{+}\right\}.$$

In this way, the principal implicitly determines all radii considered below. This is done separately for issues where the sequence $C{\left(R_{i,0}\right)}_{i=1}^{n}\subset O$ visualises the importance of individual issues. The guiding principle here is

*If the issue* $f_{i}$ *is more important than the issue* $f_{k}$ *then* $V\left(C\left(R_{i,0}\right)\right)\;>$ $V\left(C\left(R_{k,0}\right)\right)$

where the symbol V(C(ϕ)) denotes the size of the circle C(ϕ).

Then, for each list $X_{i}$ of predefined options, principal separately visualises the preferences between options by the sequence $C{\left(R_{i,j}\right)}_{j=1}^{m_{i}}\subset O$. The rule is that:

*If the option*$x_{i,j}$*is better than the option*$x_{i,k}$*then*$V\left(C\left(R_{i,j}\right)\right)>V\left(C\left(R_{i,k}\right)\right)$.

Therefore, we can consider each sequence ${\left(V\left(C\left(R_{i,j}\right)\right)\right)}_{j=1}^{m_{i}}$ as relative utility determined for options assigned to the issue $f_{i}\;\left(i=1,2,\ldots ,n\right)$. In practice, the sequence ${\left({\left(R_{i,j}\right)}_{j=0}^{m_{i}}\right)}_{i=1}^{n}$ of all applied radii is usually unknown to us. However, for the purposes of theoretical discussion only, we assume that the radii used are known to us.

**Figure 1:**
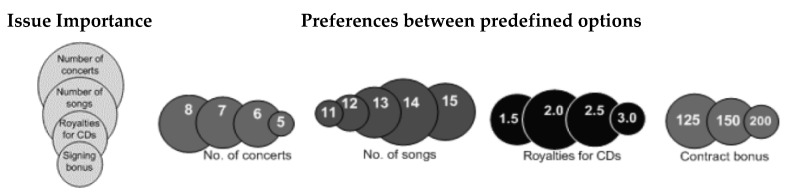
Visualisation of the principal’s preferences.

**Example** **2.** *In the negotiation described in Example 1, the management of the broadcasting company is a principal to the negotiation agent and visualises its preferences using circles, as shown in Figure 1*.

Table 2 shows the radii of the circles used for the principal’s visualisation.

Example 2 shows that the issues’ importance and preferences may be visualised using different scales. We can only notice that each circle $C\left(R_{i,j}\right)\;\left(i=1,2,\ldots ,n;j=0,1,2,\ldots ,m\right)$ is uniquely represented by its radius $R_{i,j}$. For the needs of the scoring systems built in Inspire, these circles are standardised separately for visualisation ${\left(C\left(R_{i,0}\right)\right)}_{i=1}^{n}$ of issue importance and for relative utilities of options ${\left(V\left(C\left(R_{i,j}\right)\right)\right)}_{j=1}^{m_{i}}\left(i=1,2,\ldots ,n\right)$. We standardise the issue importance visualisation in a similar way, in which the weights are calculated

(10) $$\forall_{i-1,2,\ldots ,n}:\;r_{i,0}=\frac{R_{i,0}}{\sum_{q=1}^{n}R_{q,0}},$$

The standardisation of relative utilities describing the preferences for options may be performed using various techniques. One of them is linear max-min scaling that applies the following formula

(11) $$\forall_{i=1,2,\ldots ,n}\;\forall_{j=1,2,\ldots ,m_{i}}:r_{i,j}^{\left(1\right)}=\frac{R_{i,j}-\min\left\{R_{i,q}:q=1,2,\ldots ,m_{i}\right\}}{\max\left\{R_{i,q}:q=1,2,\ldots ,m_{i}\right\}-\min\left\{R_{i,q}:q=1,2,\ldots ,m_{i}\right\}}.$$

The method based on (10) and (11) is the first standardisation method implemented by Kersten [2] in the Inspire negotiation system. Therefore, we call it INSPIRE 1.

**Example** **3.** *Table 3 shows the circles radii standardised using INSPIRE 1 for visualisation of the principal’s preferences for Mosico agents.*

The above example implies some more general conclusions. Let us compare the standardised relative utilities assigned to issues: $f_{3}=$ “Royalties” and $f_{4}=$ “Bonus”. In each of these issues, the relative utilities of the options $x_{3,3}=$ “2.5%” and $x_{4,1}=$ “$125,000” are visualised by the same circles. Moreover, the worst option $x_{3,4}=$ “3%” is visualised by circle greater than circle visualising the worst option $x_{4,3}=$ “$200,000”. In the INSPIRE 1 method, the relative utility of options $x_{4,3}$ is less than the relative utility $x_{4,1}$. According to common sense, the relative utility of options $x_{3,4}$ should be greater than the relative utility of options $x_{4,3}$. But in INSPIRE 1, the relative utility of options $x_{3,4}$ is equal to the relative utility of options $x_{4,3}$. This is a significant drawback of the INSPIRE 1 method.

For this reason, we propose the second variant of the standardisation of relative utilities that will use max scaling, which we name INSPIRE 2. In this method, the standardised radii for visualisations of preferences between predefined options will be calculated in the following way

(12) $$\forall_{i=1,2,\ldots ,n}\;\forall_{j=1,2,\ldots ,m_{i}}:r_{i,j}^{\left(2\right)}=\frac{R_{i,j}}{\max\left\{R_{i,q}:q=1,2,\ldots ,m_{i}\right\}}.$$

**Example** **4.** *Table 4 shows the circles radii standardised with the use of INSPIRE 2 for visualisation of the principal’s preferences*.

Let us note that in INSPIRE 2, the relative utility of options $x_{3,4}$ is greater than the relative utility of options $x_{4,3}$. This is a significant advantage of the INSPIRE 2 method.

When the scoring system is built, an agent uses the preference information visualised by the principal and tries to map them into the system of cardinal scores comparing the circle sizes. However, understanding the phrase “circle size” depends on the applied pragmatics of the natural language. Therefore, the linguistic variable “circle size” is imprecise. Brinton [66] recognised some problems with using circles as a tool for information presentation. The guiding principle of the method considered by him was that the greater utility of a characterised object implies the larger size of the representing circle. He showed that circle sizes evaluated by circle radius or by circle area make the reader misperceive the relative utility of the objects described by these circles. Brinton noticed that:

- *comparison between radii causes overestimation of the relative utility of the worse object;*
- *comparison between areas causes underestimation of the relative utility of the worse object.*

Many authors confirm these observations [22,67,68,69]. They conclude that circles’ relative sizes are misperceived, and these mistakes are systematic (see in [22]). Therefore, they propose such a formula of the “circle relative size” function, which allows the “psychologically correct” circle sizes to be calculated. Their proposition implies that the “circle size” function $V\left(\cdot |\gamma \right):O\to {\mathbb{R}}_{0}^{+}$ is given by the identity

(13) $$V\left(C\left(r\right)|\gamma \right)=\alpha \cdot r^{\gamma },$$

where $\alpha \in {\mathbb{R}}^{+}$ is a size of benchmark circle C(1). The exponent γ characterises an agent’s understanding of the circle size. Some empirical studies prove that γ varies from 1.6 to 1.82 (see [22,67]).

In the Inspire system, during the second step of the prenegotiation preparation phase, an agent assesses the circle size $V\left(C\left(R_{i,j}\right)\right)$ by value $V_{i,j}\in {\mathbb{R}}_{0}^{+}\;\left(i=1,2,\ldots ,n;j=0,1,2,\ldots ,m_{i}\right)$. According to the studies mentioned above, agents may use different scales to assess the circles’ size. Moreover, in determining the relative utility, each unknown radius $R_{i,j}$ maybe replaced by any value $V_{i,j}$. For these reasons, we standardise agent answers in the same way as the radii of the circles drawn by the principal. Therefore, the issue weights are calculated in the following way

(14) $$\forall_{i=1,2,\ldots ,n\;}:v_{i,0}=\frac{V_{i,0}}{\sum_{q=1}^{n}V_{q,0}}.$$

Then the standardised description of preferences between predefined options is determined for the INSPIRE 1 method in the following way

(15) $$\forall_{i=1,2,\ldots ,n}\;\forall_{j=1,2,\ldots ,m_{i}}:v_{i,j}^{\left(1\right)}=\frac{V_{i,j}-\min\left\{V_{i,q}:q=1,2,\ldots ,n_{i}\right\}}{\max\left\{V_{i,q}:q=1,2,\ldots ,n_{i}\right\}-\min\left\{V_{i,q}:q=1,2,\ldots ,n_{i}\right\}},$$

while for the INSPIRE 2 method, the following formula is used

(16) $$\forall_{i=1,2,\ldots ,n}\;\forall_{j=1,2,\ldots ,m_{i}}:v_{i,j}^{\left(2\right)}=\frac{V_{i,j}\;}{\max\left\{V_{i,q}:q=1,2,\ldots ,n_{i}\right\}\;}.$$

For any option $x_{i,j}\;\left(i=1,2,\ldots ,n;j=1,2,\ldots ,m_{i}\right)$, its absolute utility $U\left(x_{i,j}\right)$ depends on its relative utility $V\left(C\left(R_{i,j}\right)\right)$ and on the importance of the issue $f_{i}$. Therefore, we can assume that its absolute utility $U\left(x_{i,j}\right)$ is directly proportional to the relative utility $V\left(C\left(R_{i,j}\right)\right)$ and to the weight $v_{i,0}$ of issue $f_{i}$. As a result, for the fixed INSPIRE Q method (Q=1,2) we transform the agent’s rating in the following way

(17) $$\forall_{Q=1,2}\forall_{i=1,2,\ldots ,n}\forall_{j=1,2,\ldots ,m_{i}}:u_{i,j}^{\left(Q\right)}=v_{i,0}\cdot v_{i,j}^{\left(Q\right)}.$$

The series of absolute utilities determined from formula (17) is used to build the scoring function (8). The results of the discussion in Examples 3 and 4 show that scoring functions obtained using INSPIRE 1 and INSPIRE 2 methods would be different.

## 4. The Prenegotiation Experiment

We organised a prenegotiation experiment to prove that the representations of the scoring system reflecting predefined principal’s preferences differ for various agents, which justifies the need for the fuzzy approach in representing the scoring function. In the experiment, we used a negotiation case from the Inspire negotiation system described in Examples 1 and 2. The negotiation template consisted of four issues and lists of options (see Table 1) that allow building 240 packages. All the respondents played the role of Mosico agents. The participants were asked to interpret the preference information of their agent (a visualisation through circles) and determine the scoring system for the Mosico party. While determining the scoring systems, they follow the prenegotiation protocol that is classically implemented in Inspire, i.e., they assigned the cardinal ratings to the options and issues according to their individual understanding of the differences in circles’ sizes using crisp values $V\left(C\left(R_{i,j}\right)\right)$.

The experiment was conducted in the form of an in-class survey. The respondents were the bachelor and master students of four Polish universities, and the experiment was a part of their courses in decision making and decision support. We received 141 completed questionnaires. Of the 141 respondents, there were 83 males (almost 59%). It occurred that the respondents used different standardisation methods—presumably based on their earlier experience with MCDA methods—when providing the cardinal evaluation of circle sizes, using either formula (15) or (16). Therefore we divided them into two groups. The first group consisted of 38 respondents preferring the INSPIRE 1 method, and the second—103 respondents preferring the INSPIRE 2 method to standardise their scores. The answers obtained from the respondents from the first and second groups were used to determine their utilities in the way specified in the INSPIRE 1 and INSPIRE 2 methods.

During the analysis of the prenegotiation experiment data, we compared the assessment of circle size determined by l>1 respondents within each group. The kth respondent assessed the circle size $V\left(C\left(R_{i,j}\right)\right)$ by value $V_{i,j,k}\in {\mathbb{R}}_{0}^{+}\;\left(i=1,2,\ldots ,n;j=0,1,2,\ldots ,m_{i};k=1,2,\ldots ,l\right)$. Let us take into account the fixed INSPIRE Q method (Q=1,2). Then we transform the kth respondent’s rating using the following formula:

(18) $$\forall_{Q=1,2}\;\forall_{i=1,2,\ldots ,n}\forall_{j=1,2,\ldots ,m_{i}}\;\forall_{k=1,2,\ldots ,l}:u_{i,j,k}^{\left(Q\right)}=v_{i,0,k}\cdot v_{i,j,k}^{\left(Q\right)},$$

where

(19) $$v_{i,0,k}=\frac{V_{i,0,k}}{\sum_{q=1}^{n}V_{q,0,k}}.$$

(20) $$v_{i,j,k}^{\left(1\right)}=\frac{V_{i,j,k}-\min\left\{V_{i,q,k}:q=1,\ldots ,m_{i}\right\}}{\max\left\{V_{i,q,k}:q=1,\ldots ,m_{i}\right\}-\min\left\{V_{i,q,k}:q=1,\ldots ,m_{i}\right\}\;},$$

(21) $$v_{i,j,k}^{\left(2\right)}=\frac{V_{i,j,k}}{\max\left\{V_{i,q,k}:q=1,\ldots ,m_{i}\right\}}.$$

The values $u_{i,j,k}^{\left(Q\right)}$ are interpreted as the kth respondent’s utility (rating) of option $x_{i,j}$. In our experiment, we evaluated the respondent’s ratings in the following way. For each option $x_{i,j}\;\left(i=1,2,\ldots ,n;j=1,2,\ldots ,m_{i}\right)$ and for each INSPIRE Q method (Q=1,2), we calculated the relative dispersion index given by the formula

(22) $$\delta_{i,j}^{\left(Q\right)}=\frac{\max\left\{u_{i,j,k}^{\left(Q\right)}:k=1,2,\ldots ,l\right\}-\min\left\{u_{i,j,k}^{\left(Q\right)}:k=1,2,\ldots ,l\right\}}{l^{-1}\cdot \sum_{k=1}^{l}u_{i,j,k}^{\left(Q\right)}}.$$

The results obtained are presented in Table 5.

Let us note that the calculated values of the relative dispersion index vary from 60% to 424%. The conclusion is obvious; utility ratings given by respondents reveal significant differences in interpreting the principal’s preference information. This shows that operating with the scoring systems based on real numbers (crisp values of utilities) may be unjustified as they cannot handle the imprecision that is associated with the preference information vaguely visualised by means of circles.

## 5. Fuzzy Scoring System

Given the results presented in the previous section, any agent who prepares the scoring system should consider the fact that the ratings he provides may not precisely address the principal’s true preferences. The agents may be biased by their information processing style or number sense, or other behavioural factors. For this reason, we will assume that the agent asks l>1 different respondents to rate the principal’s preferences to provide them with potential information of the range of possible understanding of these preferences. During these consultations, the kth respondent assesses the circle size $V\left(C\left(R_{i,j}\right)\right)$ by value $V_{i,j,k}\in {\mathbb{R}}_{0}^{+}\;\left(i=1,2,\ldots ,n;j=0,1,2,\ldots ,m_{i};k=1,2,\ldots ,l\right)$.

Let us take into account the fixed INSPIRE Q method (Q=1,2). Then, by using formulas (18)–(21), we transform the kth respondent’s rating into utility rating $u_{i,j,k}^{\left(Q\right)}$. In this way, we determine the set

(23) $$U_{i,j}^{\left(Q\right)}=\left\{u_{i,j,k}^{\left(Q\right)}:k=1,2,\ldots ,l\right\}\subset \mathbb{R}.$$

The set $U_{i,j}^{\left(Q\right)}$ contains all information about the utility $U\left(x_{i,j}\right)$ of the option $x_{i,j}\;\left(i=1,2,\ldots ,n;j=1,2,\ldots ,m_{i}\right)$ determined in the context of INSPIRE Q method (Q=1,2). It is the only quantitative information available to the agent about the utility value $\;U\left(x_{i,j}\right)$.

We have already argued that information about the utility $U\left(x_{i,j}\right)$ is ambiguous since it depends on the imprecise evaluation of the circle size. For this reason, we will evaluate any utility value $U\left(x_{i,j}\right)$ by FN. Moreover, without loss of reasoning generality, we can assume that any utility value $U\left(x_{i,j}\right)$ is evaluated by TrFN.

We propose the following method of transformation of the set $U_{i,j}^{\left(Q\right)}$ into TrFN. We divide the respondents’ ratings into three sets: underestimated utility ratings, well-estimated utility ratings, and overestimated utility ratings. We assume here that all these sets are roughly equinumerous. Therefore we apply here the notion of terciles. The elements of the sequence $U_{i,j}^{\left(Q\right)}$ are divided into these sets with the following delimitation:

- the least underestimated utility rating
  (24) $${\overset{ˇ}{u}}_{i,j}^{\left(Q\right)}=\min\left\{y:y\in U_{i,j}^{\left(Q\right)}\right\},$$
- the greatest underestimated utility rating
  (25) $${\bar{u}}_{i,j}^{\left(Q\right)}=\min\left\{y:\;\frac{\mathrm{card}\left\{z:z\leq y,\;z\in U_{i,j}^{\left(Q\right)}\right\}\;}{l}\geq \frac{1}{3},\;y\in U_{i,j}^{\left(Q\right)}\right\},$$
- the least overestimated utility rating
  (26) $${\overset{=}{u}}_{i,j}^{\left(Q\right)}=\max\left\{y:\;\frac{\mathrm{card}\left\{z:z\geq y,\;z\in U_{i,j}^{\left(Q\right)}\right\}\;}{l}\geq \frac{1}{3},\;y\in U_{i,j}^{\left(Q\right)}\right\},$$
- the greatest overestimated utility rating
  (27) $${\hat{u}}_{i,j}^{\left(Q\right)}=\max\left\{y:y\in U_{i,j}^{\left(Q\right)}\right\}.$$

The least well-estimated utility rating is equal to the greatest underestimated utility rating. The greatest well-estimated utility rating is equal to the least overestimated utility rating. Moreover, we have

(28) $${\overset{ˇ}{u}}_{i,j}^{\left(Q\right)}\leq {\bar{u}}_{i,j}^{\left(Q\right)}\leq {\overset{=}{u}}_{i,j}^{\left(Q\right)}\leq {\hat{u}}_{i,j}^{\left(Q\right)}.$$

It implies that for each INSPIRE Q method (Q=1,2) we can determine utility as a function $U^{\left(Q\right)}:\;{⋃}_{i=1}^{n}X_{i}\to F_{Tr}$ given by the identity

(29) $$U^{\left(Q\right)}\left(x_{i,j}\right)=Tr\left({\overset{ˇ}{u}}_{i,j}^{\left(Q\right)},{\bar{u}}_{i,j}^{\left(Q\right)},{\overset{=}{u}}_{i,j}^{\left(Q\right)},{\hat{u}}_{i,j}^{\left(Q\right)}\;\right).$$

Now, for each INSPIRE Q method (Q=1,2), the related scoring function (8) is given as the function ${\overset{˘}{S}}^{\left(Q\right)}:\mathbb{P}\to F_{Tr}$ determined for any negotiation package ${\bar{P}}_{p}$ by the identity

(30) $${\overset{ˇ}{S}}^{\left(Q\right)}\left({\bar{P}}_{p}\right)={⊕}_{i=1}^{n}Tr\left({\overset{ˇ}{u}}_{i,j\left(i,p\right)}^{\left(Q\right)},{\bar{u}}_{i,j\left(i,p\right)}^{\left(Q\right)},{\overset{=}{u}}_{i,j\left(i,p\right)}^{\left(Q\right)},{\hat{u}}_{i,j\left(i,p\right)}^{\left(Q\right)}\;\right)=\;=Tr\left(\sum_{i=1}^{n}{\overset{ˇ}{u}}_{i,j\left(i,p\right)}^{\left(Q\right)},\sum_{i=1}^{n}{\bar{u}}_{i,j\left(i,p\right)}^{\left(Q\right)},\sum_{i=1}^{n}{\overset{=}{u}}_{i,j\left(i,p\right)}^{\left(Q\right)},\sum_{i=1}^{n}{\hat{u}}_{i,j\left(i,p\right)}^{\left(Q\right)}\;\right),$$

where $\bar{P_{p}}\in \mathbb{P}$.

In this way, for each INSPIRE Q method, we obtain the fuzzy scoring system $\left\langle \mathbb{P},{\overset{ˇ}{S}}^{\left(Q\right)}\right\rangle$. From the formal point of view, the function ${\overset{ˇ}{S}}^{\left(Q\right)}$ may be considered as a special kind of the fuzzy SAW method [36]. The fuzzy evaluations of options constitute the fuzzy scoring system of the agent and may be used for the evaluation of the negotiation packages.

**Example** **5.** *For results obtained in the prenegotiation experiment described in Section 4, the utility function ${\overset{˘}{S}}^{\left(Q\right)}:\mathbb{P}\to F_{Tr}$is presented in Table 6*.

This utility function may be applied for the comparison of different 28 680 pairs of negotiations packages. For better readability of the remainder of the example, we restrict our considerations to the negotiating packages’ set ${\mathbb{P}}^{\nabla }=\left\{{\bar{P}}_{1},\;{\bar{P}}_{2},\;{\bar{P}}_{3},\;{\bar{P}}_{4},\;{\bar{P}}_{5},{\bar{P}}_{6}\right\}\subset \mathbb{P}$ described in Table 7. The offers’ global utility values are determined from formula (30) and presented in Table 7 for INSPIRE 1 and in Table 8 for INSPIRE 2.

Negotiation package preference on the set ${\mathbb{P}}^{\nabla }$ made through formula (5) is presented in Table 9 and Table 10 for INSPIRE 1 and INSPIRE 2, respectively.

The membership functions $\psi_{\max{\mathbb{P}}^{\nabla }}:{\mathbb{P}}^{\nabla }\to \left[0,1\right]$ determined for Q=1,2 by (6) are presented in Table 11. These membership functions determine the subsets of nondominated negotiation packages.

Let us observe that the results obtained for groups using methods INSPIRE 1 and INSPIRE 2 differ in values of membership functions. However, both scoring systems indicate some offers as non-dominated in the highest degree of 1, which the agents can use in the forthcoming negotiation as opening offers.

## 6. Discussion

Let us discuss the benefits of using the approach proposed in the previous section by the agents in the negotiation process. As shown in Section 4, individual respondents varied in interpreting the preference information significantly. Using the single scoring system of any particular respondent could make an agent mislead by the former’s individual behavioural capabilities and biases. For instance, when we compare three selected recommendations of the respondents (R1, R2, and R3) towards how the scoring systems should look like, we would obtain the following results as shown in Table 12.

As can be seen, the interpretation of the relative circle size differs for our three respondents as well as when compared to the ratings that could be obtained for the principal by direct mapping the circles’ radiuses (see Table 3) into the ratings. Note that not only do the differences in sizes affect the scoring systems but so do some biases that occur when processing the non-monotonous preferences (see $u_{3,1}^{\left(1\right)}$ and $u_{3,3}^{\left(1\right)}$ for principal and R3). These differences may easily lead to different rankings of the final 240 packages, including those six ones considered in the previous section. The global utilities for offers from ${\mathbb{P}}^{\nabla }$ and selected respondents from INSPIRE 1 group are shown in Table 13.

It is worth noting that the package ${\bar{P}}_{6}$ was identified as the best (dominating others) by all 38 respondents in the INSPIRE 1 group. Using the respondents’ individual recommendations will lead the agent to contradictory conclusions on the selection of the non-dominated offer that could be submitted to the negotiation table during the subsequent rounds of actual negotiations. While it is clear that the opening offer should be ${\bar{P}}_{6}$ (R1, R2, and R3 recommend it as the best one), in the further negotiation rounds, the agent could take different decisions following recommendations of R1 or R2. For the former, in the second round of negotiation, the agent would submit the package ${\bar{P}}_{3}$, while using the scoring system of R1—${\bar{P}}_{2}$ should be submitted. A similar situation occurs in the fourth round of negotiations (assuming ${\bar{P}}_{6},\;{\bar{P}}_{2}$ and ${\bar{P}}_{3}$—as three best in a row—were previously rejected). Following the recommendations of either R1 or R2, offer ${\bar{P}}_{5}$ should be submitted, while R3 suggests using ${\bar{P}}_{1}$. Even more conflicting recommendations occur in the fifth round when all three respondents recommend different packages to be submitted. Similar differences occur for the INSPIRE 2 group (Table 14).

Hence, following the recommendation of a single selected respondent does not make the agent aware of the imprecision of this single evaluation resulting from the individual perception and cognitive capabilities of that respondent and ambiguity of the evaluation of the offers linked to these behavioural issues. Consequently, the agent may be falsely convicted of the soundness and reliability of such a crisply defined scoring system by a single respondent.

Our approach that integrates the recommendations of various respondents into one scoring system allows an agent to have a deeper insight into the whole scope of potential interpretations. It aggregates the individual opinions on the principal’s preferences into fuzzy evaluations, which still convey the interpretational nuances. As can be read from Table 11, for the INSPIRE 1 group, the package ${\bar{P}}_{6}$ received the highest $\psi_{\max}$ value (equal to 1), which univocally recommends it to be submitted as first (it is not worse than any other package from our set ${\mathbb{P}}^{\nabla }$). Then, packages ${\bar{P}}_{2}$ and ${\bar{P}}_{3}$ are identified with the second highest $\psi_{\max}$ values, yet the former has a slightly higher one. Therefore, ${\bar{P}}_{2}$ should be submitted as an offer in the second round and ${\bar{P}}_{3}$ in the third one, if the counterpart would reject the offers from previous rounds. However, if the negotiation protocol (e.g., implemented in any software system that supports this negotiation) does not allow reconsideration of previously rejected offers in the forthcoming negotiation rounds, then new values of the membership functions $\psi_{\max}$ should be determined for the reduced set of offers ${\mathbb{P}}^{\nabla }\backslash \left\{{\bar{P}}_{6}\right\}$ to identify new efficient offers. Using the data from Table 9 we can easily see that eliminating ${\bar{P}}_{6}$ makes the new $\psi_{\max}$ values for packages ${\bar{P}}_{2}$ and ${\bar{P}}_{3}$ the same and equal to 1. Hence, both packages could be considered equally good and could be submitted in the second negotiation round as the alternative compromise proposals.

Finally, the same $\psi_{\max}$ values obtained by packages ${\bar{P}}_{1},\;{\bar{P}}_{4}$, and ${\bar{P}}_{5}$ (equal to 0) identify them as similarly attractive. Given many different opinions of the respondents regarding these packages (seemingly often conflicting), our system does not recommend any of them as being better or worse than others, at least when all six offers from ${\mathbb{P}}^{\nabla }$ are considered feasible. Thus, the agent in the next rounds of negotiation may consider them as efficient and submit one of them as a negotiation offer to his counterpart. However, when we assume the negotiation protocol that eliminates from the set of feasible offers the ones rejected on the earlier stages of the negotiation process, iterative recalculations of $\psi_{\max}$ values would be required. The series $\psi_{\max}$ values assuming rejection of the offers from the set ${\mathbb{P}}^{\nabla }$ in the subsequent negotiation rounds are presented in Table 15.

So, after proposing offers ${\bar{P}}_{2}$ and ${\bar{P}}_{3}$ that were rejected in the second round; the agent knows in round 3 that from all three remaining offers, only ${\bar{P}}_{5}$ can be univocally considered non-dominated using the group recommendation of all respondents (though some of them may individually recommend other offers, e.g., R3 would recommend ${\bar{P}}_{1}$). When ${\bar{P}}_{5}$ is rejected in the third round, ${\bar{P}}_{1}$ is identified as fully non-dominated and should be submitted in the fourth round, etc. A similar analysis may naturally be performed to INSPIRE 2 group.

## 7. Final Remarks

In our paper, we proposed an original schema for determining the scoring system out of the series of interpretations submitted to the agent by their external helpers, i.e., respondents. In view of many various crisp interpretations of the circles given by the respondents, the information aggregation mechanism was suggested that represents these options in the form of trapezoidal fuzzy numbers. In this way, our approach allowed the whole range of perceptions related to various subjective and individual cognitive processes characteristic to each respondent to be included in evaluating the principal’s preferences. The need to use a fuzzy numerical representation of the principal’s preferences was empirically justified by discussing the results obtained in the case study related to the negotiation problem implemented in the Inspire system [2,12]. The scoring function proposed in this paper allows taking into account the imprecision of preference information imparted by a principal to an agent and compare and analyse various experiments conducted in the negotiation support systems most frequently used in teaching and research.

The proposed scoring function was a special kind of fuzzy SAW function [36]. It is worth noting that some other approaches could also be used to produce such a fuzzy scoring function using either agents’ own interpretations of the principal’s preferences or the external recommendations of respondents. For instance, the fuzzy AHP procedure could be implemented [44]. This, however, would not fit the preferences elicitation protocol implemented in the Inspire system (that operates with crisp evaluations through hybrid conjoint measurement). Additionally, it would require agents or the respondents to use predefined linguistic scales with arbitrarily linked FN/TrFN. Differences in understanding the meaning of the linguistic etiquettes by the agents and various possible expectations of how such etiquettes should be associated with the cardinal evaluation provided through FN could overlap the differences in interpreting the circle sizes and negatively affect the quality of the scoring systems built this way.

From an empirical point of view, the large diversity of respondents’ opinions justifies using a fuzzy scoring function. We have also shown that such a large diversity of respondents’ opinions together with the subjectively chosen way of processing this information affect the evaluation of offers and, consequently, identification of the set of efficient negotiation packages for each agent.

It is worth noting that the approach we propose seems quite flexible. Its applicability should not be limited to situations in which circle-based visualisation is used to support preference impartation. It should not be even limited to the problems with preference visualisation at all. It seems to be applicable to every general problem in which the principal describes her preferences ambiguously. However, as we assumed imprecise visualisation through circles only, proving its generality would require additional tests and experiments.

As the approach we proposed aims to facilitate the process of preference formalisation into a form of the scoring system, further research should be conducted to verify if it is considered easy and useful. A series of experiments with the principals and agent’s respondents should be organised with the proposed prenegotiation protocol implemented in a software solution that would be a subject of evaluation. In these experiments, classic or modified tests of acceptance should be conducted, e.g., the ones that derive from the technology acceptance model [70], which would allow measuring ease of use, usefulness, and future intention of use of such prenegotiation process in real-world negotiations. Such future experiments we would like to replicate for different negotiation cases.

Further research is also required to answer how the set of efficient negotiation packages may look like when considered from the perspective of both negotiators simultaneously. This would allow checking how our approach may be used to provide the parties with symmetric negotiation support and identify the set of mutually feasible alternatives. It is also advisable to study the behavioural factors differentiating respondents’ opinions regarding the rating values, for instance, their ability to process the preferential information correctly, cognitive capabilities, or information processing style.

## Figures and Tables

**Table 1:** Example of the negotiation template.

Negotiations Issues	Lists of Predefined Options
Number of promotional concerts (per year)	5; 6; 7 or 8 concerts
Number of new songs introduced and performed each year	11; 12; 13; 14 or 15 songs
Royalties for CDs (in per cent)	1.5; 2; 2.5 or 3%
Contract signing bonus (in dollars)	$125,000; $150,000 or $200,000

**Table 2:** Original radii in preference visualisation.

Issue	Issue Importance $\left(R_{i,0}\right)$	Preferences between Options				
		$R_{i,1}$	$R_{i,2}$	$R_{i,3}$	$R_{i,4}$	$R_{i,5}$
Concerts	5.59	4.30	3.85	3.45	1.85	
Songs	4,74	2.00	2.70	3.70	4.90	4.20
Royalties	3.54	3.80	4.50	4.00	2.90	
Bonus	2.89	4.00	3.40	2.50		

**Table 3:** Standardised radii of the preference visualisation obtained with the use of INSPIRE 1.

Issue	Standardised Radii for:					
	$\mathrm{Issue}\;\mathrm{Importance}\;(r_{i,0})$	Option Importance				
		$r_{i,1}^{\left(1\right)}$	$r_{i,2}^{\left(1\right)}$	$r_{i,3}^{\left(1\right)}$	$r_{i,4}^{\left(1\right)}$	$r_{i,5}^{\left(1\right)}$
Concerts	0.3335	1	0.8162	0.6531	0	
Songs	0.2828	0	0.2414	0.5862	1	0.7582
Royalties	0.2112	0.5625	1	0.6875	0	
Bonus	0.1724	1	0.6000	0		

**Table 4:** Standardised radii of the preference visualisation obtained with the use of INSPIRE 2.

Issue	Standardised Radii for:					
	$\mathrm{Issue}\;\mathrm{Importance}\;(r_{i,0})$	Option Importance				
		$r_{i,1}^{\left(2\right)}$	$r_{i,2}^{\left(2\right)}$	$r_{i,3}^{\left(2\right)}$	$r_{i,4}^{\left(2\right)}$	$r_{i,5}^{\left(2\right)}$
Concerts	0.3335	1	0.8953	0.8023	0.4302	
Songs	0.2828	0.4081	0.5510	0.7551	1	0.8571
Royalties	0.2112	0.8444	1	0.8888	0.6444	
Bonus	0.1724	1	0.8500	0.6250		

**Table 5:** Relative Dispersion Indexes determined for the observed prenegotiation experiment.

	Options	INSPIRE 1 (l = 38)	INSPIRE 2 (l = 103)
$x_{1,1}$	8 promotional concerts	0.592	0.586
$x_{1,2}$	7 promotional concerts	0.892	0.601
$x_{1,3}$	6 promotional concerts	1.426	1.122
$x_{1,4}$	5 promotional concerts	-	2.699
$x_{2,1}$	11 new songs	-	2.255
$x_{2,2}$	12 new songs	0.953	1.534
$x_{2,3}$	13 new songs	0.864	1.173
$x_{2,4}$	14 new songs	0.333	0.599
$x_{2,5}$	15 new songs	0.424	1.035
$x_{3,1}$	1.5% royalties for CDs	1.540	1.685
$x_{3,2}$	2% royalties for CDs	0.763	1.021
$x_{3,3}$	2.5% royalties for CDs	1.081	0.926
$x_{3,4}$	3% royalties for CDs	-	2.715
$x_{4,1}$	$125,000 contract signing bonus	0.991	1.943
$x_{4,2}$	$150,000 contract signing bonus	1.090	2.615
$x_{4,3}$	$200,000 contract signing bonus	-	4.240

**Table 6:** Utility functions determined for the prenegotiation experiment.

Options	INSPIRE 1 (l = 38)	INSPIRE 2 (l = 103)
$x_{1,1}$	Tr(0.35,0.40, 0.43, 060)	Tr(0.29,0.39, 0.40, 0.53)
$x_{12}$	Tr(0.20,0.30, 0.32, 0.48)	Tr(0.23,0.30, 0.32, 0.42)
$x_{1,3}$	Tr(0.10,0.20, 0.22, 0.40)	Tr(0.13,0.20, 0.24, 0.39)
$x_{1,4}$	Tr(0.00,0.00, 0.00, 0.00)	Tr(0.00,0.07, 0.10, 0.24)
$x_{2,1}$	Tr(0.00,0.00, 0.00, 0.00)	Tr(0.00,0.06, 0.06, 0.14)
$x_{2,2}$	Tr(0.05,0.10, 0.12, 0.15)	Tr(0.05,0.12, 0.14, 0.25)
$x_{2,3}$	Tr(0.10,0.18, 0.21, 0.27)	Tr(0.08,0.18, 0.21, 030)
$x_{2,4}$	Tr(0.25,0.30, 0.30, 0.35)	Tr(0.20,0.30, 0.30, 0.38)
$x_{2,5}$	Tr(0.20,0.21, 0.25, 0.30)	Tr(0.10,0.23, 0.25, 0.34)
$x_{3,1}$	Tr(0.02,0.07, 0.09, 0.15)	Tr(0.03,0.10, 0.13, 0.23)
$x_{3,2}$	Tr(0.10,0.15, 0.20, 0.24)	Tr(0.10,0.20, 0.20, 0.30)
$x_{3,3}$	Tr(0.05,0.12, 0.15, 0.19)	Tr(0.08,0.15, 0.16, 0.21)
$x_{3,4}$	Tr(0.00,0.00, 0.00, 0.00)	Tr(0.00,0.05, 0.06, 0.14)
$x_{4,1}$	Tr(0.05,0.10, 0.10, 0.14)	Tr(0.00,0.10, 0.10, 0.21)
$x_{4,2}$	Tr(0.02,0.05, 0.05, 0.08)	Tr(0.00,0.06, 0.07, 0.19)
$x_{4,3}$	Tr(0.00,0.00, 0.00, 0.00)	Tr(0.00,0.03, 0.04, 0.16)

**Table 7:** Global utility values for negotiation packages from the set ${\mathbb{P}}^{\nabla }$ (INSPIRE 1).

No	Negotiation Packages	INSPIRE 1
${\bar{P}}_{1}$	5 concerts, 11 songs, 1.5% royalties, $125 000 contract	Tr(0.07,0.17, 0.19, 0.29)
${\bar{P}}_{2}$	7 concerts, 11 songs, 1.5% royalties, $125 000 contract	Tr(0.27,0.47, 0.51, 0.77)
${\bar{P}}_{3}$	6 concerts, 12 songs, 1.5% royalties, $150 000 contract	Tr(0.20,0.42, 0.48, 0.78)
${\bar{P}}_{4}$	5 concerts, 11 songs, 2.5% royalties, $200 000 contract	Tr(0.05,0.12, 015, 0.19)
${\bar{P}}_{5}$	5 concerts, 13 songs, 3.0% royalties, $125 000 contract	Tr(0.15,0.28, 0.31, 0.41)
${\bar{P}}_{6}$	8 concerts, 15 songs, 2.5% royalties, $200 000 contract	Tr(0.60,0.73, 0.83, 1.09)

**Table 8:** Global utility values for negotiation packages from the set ${\mathbb{P}}^{\nabla }$ (INSPIRE 2).

No	Negotiation Packages	INSPIRE 2
${\bar{P}}_{1}$	5 concerts, 11 songs, 1.5% royalties, $125 000 contract	Tr(0.03,0.33, 0.39, 0.81)
${\bar{P}}_{2}$	7 concerts, 11 songs, 1.5% royalties, $125 000 contract	Tr(0.26,0.56, 0.61, 0.99)
${\bar{P}}_{3}$	6 concerts, 12 songs, 1.5% royalties, $150 000 contract	Tr(0.21,0.48, 0.58, 1.05)
${\bar{P}}_{4}$	5 concerts, 11 songs, 2.5% royalties, $200 000 contract	Tr(0.08,0.31, 0.36, 0.76)
${\bar{P}}_{5}$	5 concerts, 13 songs, 3.0% royalties, $125 000 contract	Tr(0.08,0.40, 047, 0.89)
${\bar{P}}_{6}$	8 concerts, 15 songs, 2.5% royalties, $200 000 contract	Tr(0.47,0.80, 0.85, 1.25)

**Table 9:** Negotiation packages preference on set ${\mathbb{P}}^{\nabla }$ (INSPIRE 1).

No	${\bar{P}}_{1}$	${\bar{P}}_{2}$	${\bar{P}}_{3}$	${\bar{P}}_{4}$	${\bar{P}}_{5}$	${\bar{P}}_{6}$
${\bar{P}}_{1}$	1	0.07	0.29	1	0.62	0
${\bar{P}}_{2}$	1	1	1	1	1	0.44
${\bar{P}}_{3}$	1	1	1	1	1	0.42
${\bar{P}}_{4}$	0.86	0	0	1	0.25	0
${\bar{P}}_{5}$	1	0.46	0.66	1	1	0
${\bar{P}}_{6}$	1	1	1	1	1	1

**Table 10:** Negotiation packages preference on set ${\mathbb{P}}^{\nabla }$ (INSPIRE 2).

No	${\bar{P}}_{1}$	${\bar{P}}_{2}$	${\bar{P}}_{3}$	${\bar{P}}_{4}$	${\bar{P}}_{5}$	${\bar{P}}_{6}$
${\bar{P}}_{1}$	1	0.76	0.87	1	0.99	0.45
${\bar{P}}_{2}$	1	1	1	1	1	0.73
${\bar{P}}_{3}$	1	1	1	1	1	0.73
${\bar{P}}_{4}$	1	0.72	0.82	1	0.95	0.40
${\bar{P}}_{5}$	1	0.87	0.98	1	1	0.56
${\bar{P}}_{6}$	1	1	1	1	1	1

**Table 11:** Membership functions indicating non-dominated negotiation packages within ${\mathbb{P}}^{\nabla }$.

Negotiation Packages	$\psi_{\max\mathbb{P}}\left({\bar{P}}_{i}\right)$	
	INSPIRE 1	INSPIRE 2
${\bar{P}}_{1}$	0.00	0.45
${\bar{P}}_{2}$	0.44	0.73
${\bar{P}}_{3}$	0.42	0.73
${\bar{P}}_{4}$	0.00	0.40
${\bar{P}}_{5}$	0.00	0.56
${\bar{P}}_{6}$	1.00	1.00

**Table 12:** Absolute utilities assigned to the negotiation template elements obtained by the INSPIRE 1 method.

	$u_{1,1}^{\left(1\right)}$	$u_{1,2}^{\left(1\right)}$	$u_{1,3}^{\left(1\right)}$	$u_{1,4}^{\left(1\right)}$	$u_{2,1}^{\left(1\right)}$	$u_{2,2}^{\left(1\right)}$	$u_{2,3}^{\left(1\right)}$	$u_{2,4}^{\left(1\right)}$	$u_{2,5}^{\left(1\right)}$	$u_{3,1}^{\left(1\right)}$	$u_{3,2}^{\left(1\right)}$	$u_{3,3}^{\left(1\right)}$	$u_{3,4}^{\left(1\right)}$	$u_{4,1}^{\left(1\right)}$	$u_{4,2}^{\left(1\right)}$	$u_{4,3}^{\left(1\right)}$
**Principal**	0.33	0.27	0.22	0.00	0.00	0.07	0.17	0.28	0.21	0.12	0.21	0.15	0.00	0.17	0.10	0.00
**R1**	0.35	0.30	0.25	0.00	0.00	0.15	0.22	0.30	0.25	0.12	0.22	0.18	0.00	0.13	0.08	0.00
**R2**	0.48	0.33	0.19	0.00	0.00	0.12	0.24	0.33	0.26	0.02	0.14	0.10	0.00	0.05	0.03	0.00
**R3**	0.40	0.30	0.20	0.00	0.00	0.05	0.10	0.30	0.20	0.15	0.20	0.05	0.00	0.10	0.05	0.00

**Table 13:** Rating and ranks (in brackets) of selected offers determined for the respondents’ individual and principal’s scoring systems in INSPIRE 1.

No	Principal	R1	R2	R3
${\bar{P}}_{1}$	0.29 (5)	0.25 (5)	0.07 (6)	0.25 (4)
${\bar{P}}_{2}$	0.56 (2)	0.55 (3)	0.40 (2)	0.55 (2)
${\bar{P}}_{3}$	0.51 (3)	0.60 (2)	0.36 (3)	0.45 (3)
${\bar{P}}_{4}$	0.15 (6)	0.18 (6)	0.10 (5)	0.05 (6)
${\bar{P}}_{5}$	0.34 (4)	0.35 (4)	0.29 (4)	0.20 (5)
${\bar{P}}_{6}$	0.69 (1)	0.78 (1)	0.83 (1)	0.65 (1)

**Table 14:** Rating and ranks (in brackets) of selected offers determined for the respondents’ individual and principal’s scoring systems in INSPIRE 2.

No	Principal	R’1	R’2	R’3
${\bar{P}}_{1}$	0.61 (5)	0.13 (6)	0.20 (6)	0.40 (4)
${\bar{P}}_{2}$	0.76 (2)	0.44 (2)	0.44 (3)	0.63 (2)
${\bar{P}}_{3}$	0.75 (3)	0.33 (3)	0.46 (2)	0.58 (3)
${\bar{P}}_{4}$	0.55 (6)	0.15 (5)	0.22 (5)	0.34 (6)
${\bar{P}}_{5}$	0.67 (4)	0.24 (4)	0.36 (4)	0.38 (5)
${\bar{P}}_{6}$	0.87 (1)	0.80 (1)	0.87 (1)	0.83 (1)

**Table 15:** Membership functions indicating non-dominated negotiation packages within subsequent ${\mathbb{P}}_{t}^{\nabla }$ in the *t*th negotiation round for the INSPIRE 1 group.

Negotiation Packages	$\psi_{\max\mathbb{P}}\left({\bar{P}}_{i}\right)$			
	Round 1	Round 2	Round 3	Round 4
${\bar{P}}_{1}$	0.00	0.07	0.62	1.00
${\bar{P}}_{2}$	0.44	1.00	X	X
${\bar{P}}_{3}$	0.42	1.00	X	X
${\bar{P}}_{4}$	0.00	0.00	0.25	0.86
${\bar{P}}_{5}$	0.00	0.46	1.00	X
${\bar{P}}_{6}$	1.00	X	X	X

## Data Availability

Not applicable.
